# Genetic diversity and demographic instability in *Riftia pachyptila *tubeworms from eastern Pacific hydrothermal vents

**DOI:** 10.1186/1471-2148-11-96

**Published:** 2011-04-13

**Authors:** D Katharine Coykendall, Shannon B Johnson, Stephen A Karl, Richard A Lutz, Robert C Vrijenhoek

**Affiliations:** 1 USGS-Leetown Science Center, Aquatic Ecology Branch, Kearneysville, WV, USA; 2 Monterey Bay Aquarium Research Institute, Moss Landing, CA, USA; 3 Hawai`i Institute of Marine Biology, University of Hawai`i, Mānoa, Kāne`ohe, HI, USA; 4 Institute of Marine and Coastal Sciences, Rutgers University, New Brunswick, NJ, USA

**Keywords:** Annelida, Polychaeta, Siboglinidae, vent, metapopulations

## Abstract

**Background:**

Deep-sea hydrothermal vent animals occupy patchy and ephemeral habitats supported by chemosynthetic primary production. Volcanic and tectonic activities controlling the turnover of these habitats contribute to demographic instability that erodes genetic variation within and among colonies of these animals. We examined DNA sequences from one mitochondrial and three nuclear gene loci to assess genetic diversity in the siboglinid tubeworm, *Riftia pachyptila*, a widely distributed constituent of vents along the East Pacific Rise and Galápagos Rift.

**Results:**

Genetic differentiation (*F*_*ST*_) among populations increased with geographical distances, as expected under a linear stepping-stone model of dispersal. Low levels of DNA sequence diversity occurred at all four loci, allowing us to exclude the hypothesis that an idiosyncratic selective sweep eliminated mitochondrial diversity alone. Total gene diversity declined with tectonic spreading rates. The southernmost populations, which are subjected to superfast spreading rates and high probabilities of extinction, are relatively homogenous genetically.

**Conclusions:**

Compared to other vent species, DNA sequence diversity is extremely low in *R. pachyptila*. Though its dispersal abilities appear to be effective, the low diversity, particularly in southern hemisphere populations, is consistent with frequent local extinction and (re)colonization events.

## Background

Demographic instability influences the distribution of genetic diversity within and among discrete colonies of a widely distributed species [1,2]. Local extinction and (re)colonization events are expected to reduce the overall effective size of a metapopulation thereby limiting its capacity to retain genetic variation [3]. Furthermore, genetic drift contributes significantly less to geographical differentiation if the average persistence time of colonies is less than the time it takes to fix neutral alleles [4]. The number of colonists, their sources (i.e. migrant vs. propagule pools), and ongoing rates of gene flow all affect the degree to which demographic instability affects geographical differentiation [5-7]. Yet, these parameters are rarely known; instead they are commonly inferred from statistical analyses of genealogical variation that is assumed to be selectively neutral [8]. For a variety of practical reasons, recent studies have relied mostly on information from mitochondrial DNA variation, although the assumption of selective neutrality has been debated [9-11]. Independent genealogies involving nuclear genes are needed to distinguish the effects of idiosyncratic processes such as mitochondrial selective sweeps from demographic processes that leave genome-wide signatures [12,13].

**Figure 1:**
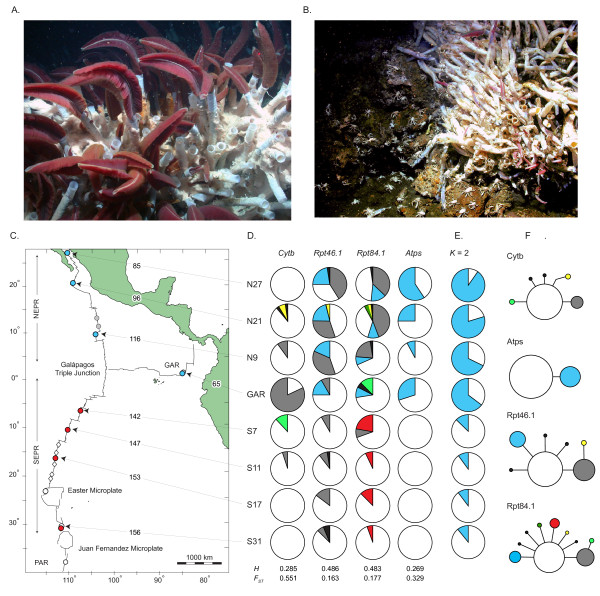
**Habitat instability and genetic diversity in *R. pachyptila***. (A) A healthy patch of tubeworms at the N27 locality. (B) An adjacent senescent patch on a rust-colored sulfide mound covered with numerous scavengers, the galatheid squat lobster *Munidopsis subsquamosa*. (C) *Riftia pachyptila *samples: blue and red dots indicate northern and southern sample locations; gray dots indicate active vents known to host *R. pachyptila *but not included in present analyses; white dots denote active vents that did not host substantial *R. pachyptila *colonies during the times of our expeditions; and white diamonds denote vents that did not support *R. pachyptila *colonies. Tectonic spreading rates are indicated along arrows for each location. (D) Allelic frequencies at four loci. Colors are coded to adjacent haplotype networks (black wedges are singletons). (E) Structure plots for the mean probability of assignment of individuals (*Q *values) to the northern cluster (blue) versus the southern cluster (white). (F) Haplotype networks for six genetic markers. Area of circle is proportional to the frequency of each haplotype, and straight lines denote single nucleotide differences.

The communities of marine animals that occupy deep-sea hydrothermal vents provide unusual opportunities to study dynamic metapopulation processes [14-16]. Vent communities are supported by chemosynthetic bacteria that oxidize volcanic gases (e.g., H_2_S and CH_4_) concentrated in the hydrothermal effluents. Adults of the iconic tubeworm *Riftia pachyptila *(Polychaeta: Siboglinidae), for example, are nourished entirely by sulfur-oxidizing endosymbiotic bacteria, linking their lives to the tempo of disturbances that create and destroy their habitats (Figure 1A-B). Eastern Pacific vents (Figure 1C) that host *R. pachyptila *are particularly ephemeral, persisting for a few years to several decades before fluid conduits are blocked, magma supplies shift, or lava flows extirpate local communities [17]. Earthquakes can open fluid conduits, re-activating old vents, and magmatic eruptions spawn new vents [18]. Studies of vent community succession [18] revealed that *R. pachyptila *is among the first species to colonize a new vent once suitable conditions are established. Within two years its numbers can grow to several thousand adult individuals, but changes in vent flow, or overgrowth by mytilids can lead to its replacement as the dominant species -- a process that can take months to years depending on the location. The frequency of habitat turnover varies with tectonic spreading rates [19]. Tectonic spreading at Eastern Pacific vents (Figure 1C) varies from moderate rates (65 mm/yr) along the Galápagos Rift (GAR) to fast rates (85-116 mm/yr) along the northern East Pacific Rise (NEPR) and superfast rates (142-158 mm/yr) along the southern EPR (SEPR). Cycles of habitat disturbance associated with these events are expected to favor organisms with rapid individual growth rates, early reproduction, and effective dispersal capabilities [20].

Active vents along the EPR and GAR are restricted to axial grabens (rift valleys) that are expected to function as dispersal corridors for the larvae of species like *R. pachyptila *[21]. The distances between active vents vary from a few kilometers along a ridge segment to 100s of kilometers between contiguous segments (Figure 1C). A one-dimensional stepping-stone model [22] provides a reasonable starting hypothesis for connectivity among linear habitats such as these, but population genetic studies provide limited support for this configuration, partly due to under-sampling of populations and insufficiently polymorphic genetic markers [23]. However, *R. pachyptila *was first among the few species that exhibited evidence for stepping-stone dispersal [24]. Estimates of gene flow (*Nm*), inferred from pairwise *F*_*ST*_'s for allozyme differentiation, declined with geographical distances among NEPR and GAR populations. Based on larval durations and predominant currents along the NEPR, *R. pachyptila *is expected to have mean larval dispersal distances of 100 to 200 km [17,21]. A subsequent phylogeographic survey examined additional samples from the SEPR axis, but found no evidence for stepping-stone dispersal due to a near absence of sequence variation in mitochondrial Cytochrome oxidase I, (*COI*) [25]. A number of co-distributed species of annelids and mollusks have been similarly examined, and they all exhibit substantial *COI *variation [25-27]. Their mitochondrial haplotype diversities range from a minimum of 0.331 in *R. pachyptila *to a maximum of 0.960 in another annelid, with a mean of 0.615 across 11 species [summarized in Figure five of reference 15]. Examinations of nuclear allozymes, AFLPs and DNA microsatellites from limited portions of *R. pachyptila*'s range [24,28,29] suggests that its low *COI *variation may be anomalous, resulting perhaps from a mitochondrial selective sweep. Nevertheless, allozymes, AFLPs and microsatellites exhibit mutation rates that do not compare to mitochondrial DNA sequences, and they might experience different selective constraints. A study including nuclear DNA sequences with sufficient numbers of individuals sampled from *R. pachyptila's *known geographic range has yet to be performed.

Testing selective sweep versus population bottleneck hypotheses requires an examination of multiple independent genetic markers [13]. To that end, we examined DNA sequences from three independent nuclear loci and another mitochondrial locus, Cytochrome *b *(*Cytb*), from *R. pachyptila*. Samples were collected from known vents along the GAR, NEPR and SEPR axes (Figure 1C). First, we tested the selective sweep hypothesis by assessing whether mitochondrial diversity within and among populations is idiosyncratically low. Second, we examined whether population structure is consistent with a linear stepping stone model, or if more complex models of subdivision are justified. Finally, we assessed whether demographic instability associated with tectonic spreading is correlated with reduced levels of genetic diversity.

## Methods

### Samples

Expeditions conducted between 1998 and 2005 explored and sampled hydrothermal vents at 19 locations along the NEPR, SEPR and GAR axes (Figure 1C). The samples examined in this study were obtained from nine locations (Table 1). Worms were harvested with robotic manipulators on the human occupied vehicle (HOV) *Alvin *(Woods Hole Oceanographic Institution, WHOI) or the remotely operated vehicle (ROV) *Tiburon *(Monterey Bay Aquarium Research Institute, MBARI). Samples were placed in an insulated container with ambient seawater. Upon recovery at the surface, whole worms were stored in 2°C filtered seawater prior to tissue sampling. A small sample of vestimentum, a tissue that is normally devoid of endosymbiotic bacteria, was frozen at -70°C or preserved in 95% EtOH.

**Table 1:** Coordinates and depths for *Riftia pachyptil a *samples.

Locality	**Lat.**^**1**^	**Lon.**^**1**^	Depths (m)	**Dive numbers**^**2**^	Sampling dates	***N***^**3**^
GAR	0.8	-86.2	2460-2461	A2005-21, A2224	4-1998, 5-1990	20
N27	27.0	-111.4	1993-2019	A3517-21, T549-51	1-2000, 4-2003	33
N21	20.8	-109.1	2577-2615	A2230-33, T556-7	6-1990, 6-2003	54
N9	9.8	-104.2	2550-2518	A2351, A3540	4-1991, 4-2000	19
S7	-7.4	-104.8	2746-2747	A3320-21	12-1998	19
S11	-11.3	-110.5	2791	A3323	12-1998	22
S17	-17.4	-113.2	2582-2599	A3328-30	12-1999	22
S23	-23.8	-115.5	2649	A4097	4-2005	1
S32	-31.2	-111.9	2333-2338	A3338-41	1-1999	9

^1 ^Positive number indicates north latitudes, negative numbers south latitudes and west longitudes.

^2 ^Submersible dive numbers: A = HOV *Alvin*; and T = ROV *Tiburon*.

^3 ^Sample size.

### DNA purification, PCR conditions and DNA sequencing

Genomic DNA was extracted using the Qiagen DNeasy kit, following manufacturer's protocols (Qiagen, Inc. Valencia, CA). The primers, optimal annealing temperatures, and PCR conditions are available in Additional file 1. We used two PCR programs: (*i*) initial denaturation temperature of 94°C for 10 min followed by 35 cycles of 94°C for 1 min, 55°C for 1 min and 72°C for 1 min with a single final extension of 72°C for 7 min; and (*ii*) initial denaturation temperature of 94°C for 10 min followed by 35 cycles of 94°C for 2 min, 48°C for 2 min, 72°C for 2.5 min, and final extension of 72°C for 7 min. PCR products from all loci were purified by gel excision and cleaned with Montage filter units (Millipore Corp., Billerica, MA) or diluted in 50 μl sterile water and purified with Multiscreen HTS PCR 96 vacuum manifold system (Millipore Corp., Billerica, MA). Unless noted (Additional file 1), DNA sequencing employed the same PCR primers. Purified PCR products were sequenced bidirectionally on an ABI 3100 capillary sequencer using BigDye terminator chemistry (Applied Biosystems Inc., Foster, CA). Protein-coding sequences were checked for stop codons.

### Mitochondrial sequences

PCR mixtures for *Cytb *included 2.5 mM MgCl_2_, 0.4 μM of each primer, 200 μM of each dNTP, 1.25 units of *Taq *polymerase (Applied Biosystems Inc., Foster, CA), 1x PCR buffer (supplied by manufacturer), and 1 μL of DNA template in a final volume of 25 μL. *Cytb *sequences were amplified with previously published primer pairs [30] and PCR program-*i *(Additional file 1). The 377 bp amplified portion of *Cytb *corresponds to positions 10732-11108 in the *R. pachyptila *mitochondrial genome [GenBank acc. no. AY741662, deposited by 31].

### Single-copy nuclear (scn) DNAs

One of us (SAK) previously generated a series of anonymous scnDNA sequences from *R. pachyptila *(unpublished, GenBank acc. nos. U68732-U68750). We designed primers from these sequences following Karl *et al. *[methodological details in 32]. Briefly, purified total DNA from a single *R. pachyptila *individual from the GAR was digested with the restriction enzyme *Sau3a*. Fragments were sorted by size, cloned, and screened to determine genomic copy number. To design primers, we sequenced each end of the low copy clone-inserts. Nine clone-inserts were tested for their potential utility in population screening. Seven inserts generated products that could not be used because they included length variation (large indels) that confounded our scoring of individual genotypes, or they lacked sufficient polymorphism. The *Rpt46.1 *primers amplified predictable products, but internal primers were required for *Rpt84.1 *to improve its sequence quality (Additional file 1). PCR program-*i *was used for both loci. Amplification with the *Rpt46.1 *primers generated two products; the larger band was used as template for sequencing. After preliminary sequencing, many genotypes contained two polymorphic sites. To resolve the ambiguous haplotypes, we cloned the PCR products from heterozygous individuals using the standard manufacturer's protocol (Topo TA cloning kit; Invitrogen, Carlsbad, CA).

### Nuclear intron

Of seven primer pairs developed by Jarman *et al. *[33], only the *ATPSα *primers amplified a polymorphic product that could be scored in *R. pachyptila*. To improve its reliability, we designed non-degenerate internal primers (Additional file 1). The intron amplified using the same PCR recipe as above and PCR program-*ii*. Initial screening of the 473 bp product from ten individuals from nine populations revealed a single nucleotide polymorphism (A/G) at position 319 of the amplicon. Because the SNP could be recognized by the restriction enzyme *NspI*, population screening was based on restriction fragment length polymorphisms (RFLPs). Ten μl of the amplicons were suspended in a cocktail containing 1 × NE buffer-2, 10 μg/μl bovine serum albumen, 3 units of *NspI *(New England Biolabs, Ipswich, MA) and sterile water to a final volume of 15 μl, and the mix was incubated at 37°C for 1.5-11 hours. Products were separated on 3% agarose gels at room temperature for 1.5-3 hrs at 65-80 volts. Positive controls of known genotypes (from prior sequencing) were included with RFLP digestions and all gel analyses.

### Statistical analyses

We used the program Phase v. 2.1 [34,35] to reconstruct allelic haplotypes for nuclear loci using the cloned haplotypes from the double heterozygotes as a baseline. We also used the software to estimate background recombination rates per gene segment. Recombination rates were low enough to assume no effective recombination within each segment. Tcs v. 1.21 [36] was used to construct statistical parsimony networks for each locus. Arlequin 3.5 [37] was also used to estimate molecular diversity, θ_*S *_[38], from the number of segregating sites, and Fu's *Fs *[39], and to estimate gene diversity (*H*), adjusted for sample size according to Eq. 8.4 of Nei [40]. Allelic richness (*a'*) estimates were obtained by the rarefaction methods implemented in Hp-rare v. 1.0 [41]. Estimates of *F*_*IS *_and tests for Hardy Weinberg equilibrium (HWE) and linkage disequilibrium (LD) were conducted with Genepop v. 3.4 [42].

The multi-locus genotypic assignment method employed in Structure v. 2.2 [43] was used to estimate the probable number of discrete population clusters (*K*) represented by the data. We assumed correlated allele frequencies between population samples and admixed ancestries of populations. Parameter estimates for correlated allele frequencies and admixture were estimated by averaging values from preliminary simulations that varied *K *from 1 to 8. Simulations were repeated at least five times for each value of *K *(number of data partitions) and for two disparate values of λ (0.731, 2.204), a shape distribution parameter for allele frequencies, based on initial simulations. Assuming a uniform prior on *K*, we estimated a Bayes factor for each value of *K *after averaging the *ln *Pr(X/K) values between simulations. All simulations employed a burn-in value of 10^5 ^with 10^6 ^subsequent steps in the Markov Chain Monte Carlo (MCMC) sampling process.

To assess evidence for isolation-by distance (IBD) we used Arlequin 3.5 to obtain pairwise estimates of *F*_*ST*_, and the GeoDistances procedure in Le Progiciel R [44] to estimate great arc distances from geographical coordinates for the sample locations (Table 1). The Mantel test module implemented in Arlequin 3.5 was used to assess correlations between the genetic (**A**) and geographical (**B**) distance matrices. To assess possible affects of subdivision, we generated an assignment matrix (**C**) with values that equaled zero (0) if populations belonged to a cluster identified by Structure and one (1) if populations belonged to different clusters. The partial correlation (*r*_AB|C_) of **A **and **B **after accounting for **C **was obtained according to the methods of Smouse *et al. *[45] as implemented in Arlequin 3.5.

The *F*_*ST*_-outlier method [46] implemented in the program Lositan [47] was used to test for evidence of selection. The program uses empirical estimates of genic heterozygosity to simulate neutral expectations for *F*_*ST *_under an island model of migration. An iterative bisection algorithm is used to simulate data with a mean *F*_*ST *_that matches the empirical data, even when the data do not strictly adhere to island model assumptions [i.e. *F*_*ST *_≈ 1/(4*Nm *+ 1)] for neutral genes [47]. Initial simulations were conducted with 10,000 replications, assuming an infinite allele mutation model. We then conducted simulations with 40,000 replications and used the neutral mean for *F*_*ST *_to estimate its 95% confidence intervals (CIs). Empirical values were compared to this distribution and those significantly above or below the 95% CIs might be influenced by selection. We attempted to use the program Sweep_Bott [13] to distinguish population bottlenecks from a selective sweeps, but the simple star phylogenies obtained for the present genes prevented its execution.

### Geographical and geophysical methods

Seafloor spreading rates (Figure 1C) were estimated by the NUVEL-1A model implemented in the web-based calculator <http://www.ldeo.columbia.edu/~menke/plates2.html>. This kinematic plate-motion model uses tables of Euler vectors previously estimated from geomagnetic reversals [48]. Space-based geodetic methods were used to improve accuracy of the model [49]. The NUVEL-1A estimates of spreading rates are highly correlated (*r *= 0.982) with empirical measurements based on near-bottom surveys and space geodesy methods [50].

## Results

### Distribution and abundance

We explored 19 active vent fields between 27°N and 38°S latitude and obtained adequate samples of *R. pachyptila *from eight localities (Figure 1C, blue and red dots). Robust populations were observed and sampled from the NEPR localities. Northern SEPR localities S7, S11 and S17 also host robust *R. pachyptila *populations, but only one specimen was observed and sampled from S23 on the NW flank of the Easter Microplate. Nine worms were observed and sampled from S32 during four dives in 1999, and none were found when we returned there in 2005. We conducted five submersible dives at S38 and found a single individual living deeply within a recess on the vertical wall of an active chimney, but it could not be sampled before deteriorating weather conditions forced us to abandon this location.

### Polymorphism

Twenty-five alleles were identified across four polymorphic loci (Additional file 2). Sequences associated with these alleles were deposited with GenBank (acc. Nos. HQ405793-6724). For each locus, parsimony analyses produced very simple star-like genealogies with mostly one-step mutational differences from a primary (most frequent) allele (Figure 1D and 1F). Widely distributed secondary (less frequent) alleles occurred at three loci. The assignments of haplotype phases in double heterozygotes were resolved with 97% probability for *Rpt46.1 *(5 individuals) and 89-96% probability for *Rpt84.1 *(6 individuals) depending on the sample location. Haplotypes inferred with the program Phase were used in all subsequent analyses. Sequence diversity was very low in the samples for all four loci, with *π *values ranging from 0 to 0.006 and θ_*S *_values ranging from 0 to 0.004 (population values not reported).

Genotypic frequencies at the nuclear loci revealed no evidence for deviations from Hardy-Weinberg equilibrium (*F*_*IS*_, Table 2). Multi-locus tests obtained by summing *P*-values for each locus (Fisher's method) provided no indication of significant heterozygote deficiencies within population samples (8 tests, *P*_range_: 0.169-1.000). Furthermore, the grand total of these values obtained by summing across all loci and samples was not significant (*P *= 0.320). Tests for linkage disequilibrium between pairs of polymorphic nuclear loci provided no evidence for associations within populations (16 tests, *P*_range_: 0.085-1.000). Furthermore no associations existed when *P*-values from the pairwise comparisons were summed across populations (3 tests, *P*_range_: 0.484-0.971). Similarly, tests for cytonuclear disequilibrium involving *Cytb *and the three nuclear genes were non-significant within populations (13 tests, *P*_range_: 0.158-1.000) and globally across populations (3 tests, *P*_range_: 0.370-0.931).

**Table 2:** Indices of genic diversity in *Riftia pachyptila *samples.

	Locality							
**Locus**	**N27**	**N21**	**N9**	**GAR**	**S7**	**S11**	**S17**	**S32**

*Cytb n*^1^	*33*	*44*	*19*	*17*	*17*	*18*	*14*	*9*
No. alleles (*a*)	1	4	2	2	2	2	1	1
Gene diversity (*H*_*S*_)		0.211	0.189	0.337	0.214	0.108	0	0
*Rpt 46.1 n*	*56*	*98*	*38*	*36*	*36*	*38*	*34*	*16*
No. alleles (*a*)	4	4	3	3	2	3	2	3
Gene diversity (*H*)	0.674	0.660	0.647	0.446	0.157	0.198	0.258	0.242
Fixation index *(F*_*IS*_)	0.155	0.073	0.108	0.200	-0.063	-0.067	0.324	-0.037
*Rpt 84.1 n*	*62*	*104*	*38*	*40*	*36*	*42*	*40*	*18*
No. alleles (*a*)	4	6	4	4	3	2	2	2
Gene diversity (*H*)	0.632	0.650	0.494	0.272	0.475	0.136	0.224	0.111
Fixation index *(F*_*IS*_)	-0.124	-0.067	-0.068	-0.095	0.304	-0.053	-0.118	0.000
*ATPSα n*	*54*	*106*	*38*	*40*	*38*	*21*	*38*	*18*
No. alleles (*a*)	2	2	2	2	1	1	1	1
Gene diversity (*H*)	0.492	0.374	0.149	0.445	0	0	0	0
Fixation index *(F*_*IS*_)	0.098	0.092	-0.059	0.309				
								
Pr(total *F*_*IS *_= 0)	0.404	0.600	0.383	0.255	0.176	0.230	0.461	1.000
Mean of *H*_*S*_	0.450	0.474	0.370	0.375	0.211	0.110	0.121	0.082
s.d. of *H*_*S*_	0.310	0.219	0.240	0.086	0.197	0.083	0.140	0.081
Total alleles (*A*)	11	16	11	11	8	8	6	7
Allelic richness (*a'*) ^4^	2.37	2.89	2.50	2.57	1.90	1.68	1.48	1.72
Private alleles ^4^	0.14	0.65	0.08	0.40	0.23	0.11	0.00	0.25

^1 ^Number of gene sequences.

^4 ^Rarefaction estimates of mean allelic richness and mean number of private alleles per locus.

### Test of selection

Because overall differentiation, as estimated by *F*_*ST*_, varied among the four loci (0.163 for *Rpt46*.1; 0.188 for *Rpt84.1*; 0.269 for *ATPSa*; and 0.551 for *Cytb*), we used Lositan to test for evidence of natural selection. First, we determined the neutral expectation, *F*_*ST*__(nuc) _= 0.138, for the nuclear loci alone. Though all the observed *F*_*ST*__(nuc) _values were higher than the neutral mean, none fell outside of the 95% CIs. Assuming an even sex ratio and strictly maternal cytoplasmic inheritance, the neutral expectation for a mitochondrial gene is *F*_*ST*__(mt) _= 4*F*_*ST*__(nuc)_/(1 + 3*F*_*ST*__(nuc)_) [51,52]. The *F*_*ST*__(mt) _= 0.551 for *Cytb *was only slightly greater than its neutral expectation (0.533); thus, no evidence existed for directional or balancing selection having shaped geographical differentiation for these genetic markers.

### Geographical structure

We used Structure to estimate the number of population clusters (*K*) represented among 192 multilocus genotypes. For each combination of priors, *K *= 2 provided the greatest mean value for *ln *Pr(*X|K*). Bayes factors were 0.368 for *K *= 2 and zero for *K *= 1 and *K *= 3. We estimated *Q*, the probability of assignment of genotypes to the northern cluster (NEPR/GAR), and represented the mean of *Q *for each sample in Figure 1E. The means exhibited a clinal pattern between N27 and GAR, and are effectively homogeneous along the SEPR axis. A single individual sampled from S23 clustered with its neighboring samples. Hierarchical AMOVA of the eight population samples partitioned 18.8% of gene diversity between the northern and southern clusters, 9.3% within clusters, and 71.9% within samples.

**Figure 2:**
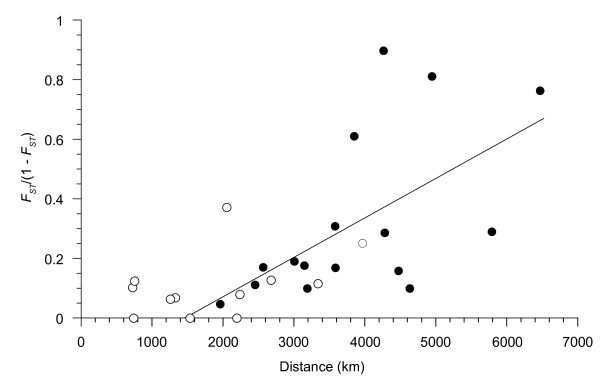
**Correlation of genetic differentiation with geographic distances among eight sample localities**. Negative values for *F*_*ST *_(Table 3) were set to zero prior to adjustment. Black dots denote contrasts between populations from the northern and southern clusters. White dots denote contrasts within clusters.

Evidence also existed for isolation-by-distance, IBD (Figure 2). Pairwise *F*_*ST*_'s (Table 3) increased significantly with geographical distances (Mantel's *r *= 0.709, *P *= 0.003); however, the comparisons between northern and southern clusters greatly influenced this relationship (black dots in Figure 2). An IBD relationship remained after exclusion of SEPR samples from the analysis (*r *= 0.896, *P *= 0.167), but the correlation was not significant. To better assess how associations with the northern and southern clusters (**C**) affected the relationship between *F*_*ST *_(**A**) and distance (**B)**, we estimated the partial correlation of **A **on **B **after accounting for **C**. A marginally significant relationship remained (*r*_AB|C _= 0.396, *P *= 0.069). It should be noted that the trans-Equatorial *F*_*ST *_between N9 and S7 (0.045) is smaller than the *F*_*ST*_'s between adjacent pairs of NEPR populations (range: 0.064-0.093). This trans-Equatorial *F*_*ST *_also is smaller than *F*_*ST*_'s between other population pairs that are about ~2000 km apart (N27 vs. N9; N9 vs. GAR; S7 vs. GAR) (Table 3).

**Table 3:** Pairwise estimates of *F*_*S*__*T *_(lower triangle) and geographic distances in km (upper triangle).

	N27	N21	N9	GAR	S7	S11	S17	S32
N27		724	2050	3963	3846	4259	4942	6465
N21	**0.093**		1327	3335	3144	3576	4276	5787
N9	**0.271**	**0.064**		2234	1957	2449	3184	4629
GAR	**0.201**	**0.104**	**0.074**		2562	3003	3581	4469
S7	**0.379**	**0.150**	0.045	**0.146**		753	1256	2673
S11	**0.473**	**0.236**	**0.101**	**0.160**	**0.111**		738	2211
S17	**0.448**	**0.223**	**0.091**	**0.145**	0.060	-0.021		1532
S32	**0.433**	**0.225**	**0.091**	**0.137**	**0.113**	-0.034	-0.015	

Bold values are statistically significant at the *α *= 0.05.

### Environmental correlates of gene diversity

**Figure 3:**
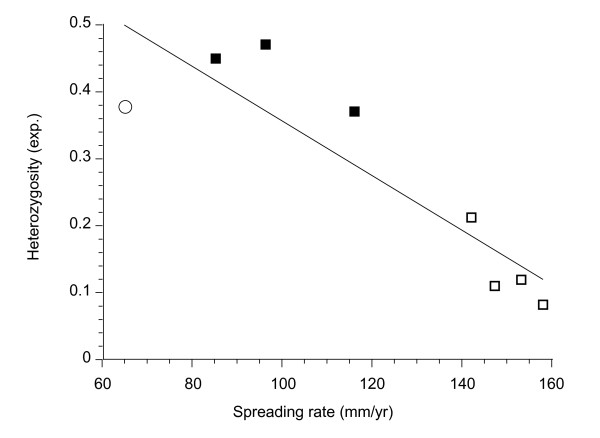
**Correlation of expected heterozygosity with tectonic spreading rates (*R***^**2 **^**= 0.788, *P = *0.0032)**. Allelic richness exhibits essentially the same relationship (*R*^*2 *^= 0.730, *P = *0.0069). Black squares are NEPR populations, white squares are SEPR samples and the white circle is the GAR population.

Heterozygosity declined strongly with tectonic spreading rate (Figure 3, *r *= -0.888), whether or not the off-axis GAR sample was included in the analysis (Table 4). Spreading rates are negatively correlated with latitude (*r *= -0.78) and the relationship is stronger with removal of the off-axis GAR sample (*r *= -0.98). Depth is relatively monotonous along the EPR axis and uncorrelated with the gene diversity indices. These correlations are clearly reflected in differences in the gene diversities between northern (NEPR/GAR) and southern (SEPR) populations, treated individually or as population clusters (Tables 2 and 5).

**Table 4:** Correlations between geographical and genetic measures for all eight samples including GAR (below diagonal) and for the seven EPR samples alone (above diagonal).

	*H*	*a'*	Private	**Spread..**.	**Lat**.	**Lon**.	Depth
Gene diversity (*H*)	-	**0.9429**	0.5195	**-0.9747**	**0.9656**	0.3306	0.0924
Allelic richness (*a'*)	**0.9420**	-	0.6906	**-0.8789**	**0.8569**	0.4267	0.0046
Private alleles	0.4779	0.6519	-	-0.4080	0.3461	0.1150	0.0115
Spreading rate	**-0.8879**	**-0.8546**	-0.5044	-	**-0.9786**	-0.1841	-0.0117
Latitude	**0.9433**	**0.8178**	0.3398	**-0.7784**	-	0.1534	0.1709
Longitude	0.3630	0.4781	0.3408	-0.6388	0.2842	-	-0.1177
Depth	0.0069	-0.1017	-0.0900	0.1883	0.1925	0.4136	-

Significant correlations (Prob. Spearman's ρ ≤ 0.01) in boldface.

**Table 5:** Genic diversity in northern and southern population clusters.

Locus	Cluster	Number of alleles *A*	Allelic diversity *H*	Nucleotide diversity *π*(%)	**Fu's *F***_***S***_	*P*
*Cytb*	North	5	0.357	0.178	-1.871	0.158
	South	3	0.098	0.040	-2.562	0.005
*Rpt46.1*	North	6	0.640	0.185	-0.476	0.449
	South	4	0.218	0.052	-2.205	0.108
*Rpt84.1*	North	9	0.621	0.208	-2.704	0.166
	South	3	0.258	0.118	0.791	0.605
*ATPSα*^1^	North	2	0.427	nd	nd	nd
	South	1	0	nd	nd	nd

^1 ^nd: not determined because data were obtained from RFLPs.

Demographic tests were initially conducted separately for each locus at each locality, but limited polymorphism in each sample provided essentially no power to reject null hypotheses. Subsequent tests were conducted for the northern and southern population clusters (Table 5). Five out of six values for Fu's *Fs *were negative, though only one was statistically significant. Mismatch distributions were all unimodal, suggesting an expanding population size, but the tests of demographic stability had very limited power due to low polymorphism.

## Discussion

*Riftia pachyptila *exhibits the lowest mitochondrial *COI *diversity observed to date among 14 species of invertebrates sampled from eastern Pacific hydrothermal vents [reviewed in 15]. Yet, the present data were not consistent with the hypothesis that a selective sweep eliminated mitochondrial diversity alone. Levels of sequence diversity were similarly low for three nuclear genes and another mitochondrial gene, *Cytb*. The shallow star-like haplotype networks exhibited by all four loci suggest that demographic factors affecting the entire genome were responsible for the reduced variation. Gene diversity indices (*H *and *a'*) for eight population samples were inversely correlated with tectonic spreading rates (Figure 3), which can be taken as surrogates for rates of habitat turnover. The lowest gene diversities occurred at southern (SEPR) localities, which are subjected to superfast spreading rates of 142-156 mm/yr. Active vents are closely spaced along the southern SEPR axis (S14-S23), but *R. pachyptila *colonies were spotty there and individuals were scarce. In contrast, higher gene diversities occurred among the northern (NEPR/GAR) localities, where spreading rates are slower, 65-116 mm/yr. Active vents are more distantly spaced along the NEPR axis, but dense *R. pachyptila *colonies occupied nearly all the active vents that we have explored along the NEPR during the past 22 years. These observations are consistent with evidence from other vent species that gene diversity is correlated with vent "occupancy" [14,15]. Species that occupy most of the active vent habitats in a region have high gene diversities compared to species with spottier distributions. Here we have found the same relationship within a species. Northern *R. pachyptila *have higher vent occupancy and gene diversity, whereas southern populations have low occupancy and low diversity. Frequent cycles of extinction and (re)colonization relative to time it takes to fix new mutations are expected to produce spotty distributions and correspondingly low gene diversity [4]. Apparently, high rates of population turnover along the southern SEPR axis has led to rapid coalescence on a small number of founders, but the origin of these founders remains a mystery.

### Population structure

Overall, differentiation among *R. pachyptila *populations meets expectations for a linear stepping-stone model. Pairwise estimates of differentiation, *F*_*ST*_/(1 - *F*_*ST*_), increased with geographical distances among the vent localities (Figure 2), particularly among the NEPR/GAR populations as previously reported for allozymes [24]. Large distances (≥2000 km) between the northern (NEPR/GAR) and southern (SEPR) population clusters greatly influenced the overall pattern, however. Hierarchical AMOVA attributed 18.8% of total gene diversity to differences between the northern and southern clusters, but this evidence for subdivision may be an artifact of sampling gaps between the two regions. Computer simulations have revealed that sampling gaps in a linear stepping-stone model can generate inflated pairwise *F*_*ST*_'s that may lead to incorrect inferences about population subdivision [23]. Here, the trans-equatorial *F*_*ST *_= 0.045 between N9 and S7 (1957 km apart, Table 3) is smaller than the *F*_*ST *_= 0.271 between the N9 and N27 along the NEPR (2050 km apart), or between N9 and GAR (2234 km apart). Even the *F*_*ST*_'s between closer pairs of NEPR samples (N27-N21 and N21-N9) were greater than the trans-equatorial pair. Estimates of pairwise migration rates (*Nm*) between adjacent EPR samples were similar, ranging from 2.0 to 5.3 after excluding the three southern SEPR samples for which *F*_*ST *_≈ 0. Nonetheless, the existence of regional alleles (blue and red in Figure 1D) suggests that dispersal across the Equator might not have achieved equilibrium with genetic drift (discussed below).

The NEPR, SEPR and GAR axes meet at the Galápagos Triple Junction (Figure 1C), which plunges to 5400 m in the 50-km wide Hess Deep. Active vents are not known from the Hess Deep proper, but video surveys of the adjacent Hess Deep Rift Valley have identified vents that host *R. pachyptila*, though they were not sampled (T. Shank 2009, pers. comm.). Further exploration of the equatorial region is warranted because we do not know why it creates a variable dispersal filter for several vent taxa [reviewed in 15]. Hurtado *et al. *[25] first noted that several annelid species exhibit variable impedance to dispersal across the Equator, and Plouviez *et al. *[26] reported that one or more historical vicariance events might have separated NEPR and SEPR populations. The palm worm, *Alvinella pompejana*, and limpets of the *Lepetodrilus elevatus *species complex exhibit evidence for isolation across the Equator, but the elevated *φ*_*ST*_'s reported by Plouviez *et al. *for other species may not be greater than expected from the sampling gaps between these axes [23]. Variable impedance across such regions results from interactions between taxon-specific dispersal modes and physical factors [15]. For example, strong deep-ocean currents sweep eastward across the EPR axis near the Equator [53]. The removal of dispersing larvae from axial troughs by these currents and the apparent scarcity of vent habitats may together impede the dispersal of some species.

### Non-equilibrium processes

Inferences about isolation-by-distance or stepping-stone models assume the constituent populations have achieved equilibrium between gene flow and genetic drift, but non-equilibrium scenarios can generate similar patterns of population structure [54]. If the Equatorial region is a contact zone between partially isolated NEPR and SEPR populations, this too could produce differentiation that resembles the product of stepping-stone dispersal, but this hypothesis fails to explain the low gene diversity in SEPR populations of *R. pachyptila *(discussed below). Magma supplies and associated tectonic and hydrothermal activities shift along ridge axes; thus, populations may be variably interconnected and disconnected by such shifts [17,55]. Numerous closely spaced vents occur between 14°and 21°S latitude (diamonds in Figure 1C), and they are very young as evidenced by recent lava flows [56,57]. Many of these vents host colonies of polychaetes, gastropods and bivalve mollusks [26,58,59], but *R. pachyptila *was rare during the expeditions we mounted between 1999 and 2005 to vents between 14°and 21°S along the SEPR. Genetic homogeneity among SEPR samples spanning 2673 km suggests that genetic drift contributes very little to differentiation among these colonies, as expected if their average persistence time is shorter than the time it takes to fix neutral alleles [4]. These ephemeral vents may offer no more than a demographic sink for *R. pachyptila*. Perhaps this region has been repeatedly colonized from more stable *R. pachyptila *sources that remain undiscovered. We searched for this species as far as 38°S latitude along a contiguous extension of the Pacific-Antarctic Ridge (PAR, Figure 1C). This latitude near the "roaring 40's" is the farthest south anyone has conducted operations with a human occupied research submersible. During five dives, we observed only a single *R. pachyptila *individual that could not be sampled before foul weather drove us from the location. If stable source populations exist elsewhere in this southern region, they would be expected to seed ephemeral SEPR vents with genetically diverse propagules, reversing the observed cline in genic diversity. Alternatively, the existence of many small (and genetically homogeneous) source populations seems unlikely given the high probability of local extinctions. Although *R. pachyptila *might exist elsewhere along the Pacific Antarctic Ridge, the present genetic evidence is consistent with colonization of the SEPR from the NEPR or GAR axes.

## Conclusion

Other studies of mitochondrial variation in vent species have reported star-like genealogies that suggest recent range expansions or demographic instability [reviewed in [15]]. Yet, the nuclear and mitochondrial gene networks from *R. pachyptila *are simpler and shallower than those found in all other vent annelids and mollusks examined to date [e.g., [25,26,60-63]]. Estimates of nucleotide diversity (*π*) for mitochondrial *COI *sequences are smaller by an order of magnitude than the nearest estimates from the other vent taxa. Comparable estimates do not exist for the present nuclear markers, but the *π *values were of the same order of magnitude as mitochondrial *Cytb *(Table 5). Although our attempts to examine indicators of demographic expansion with mismatch distributions were frustrated by the limited polymorphism, the overall lack of nuclear and mitochondrial variation itself suggests that *R. pachyptila *may be extraordinarily prone to population turnover that leads to rapid coalescence. High rates of local extinction coupled with relatively high rates of dispersal (colonization) from a limited number of source populations (i.e. propagule pool) are expected to produce the observed pattern of reduced diversity within and among disjunct colonies of a metapopulation [6,7]. Which demographic factors have helped to retain genetic diversity in the co-distributed vent species, however, remain mysteries.

## Abbreviations

ROV: remotely operated vehicle; HOV: human occupied vehicle; *COI*: cytochrome-*c*-oxidase subunit I; *Cytb*: cytochrome *b*; scnDNA: single-copy nuclear DNA; IBD: isolation-by-distance; SEPR: southern East Pacific Rise; NEPR: northern East Pacific Rise; GAR: Galápagos Rift; AFLP: amplified fragment length polymorphism.

## Authors' contributions

RAL and RCV conceived of the project and led oceanographic expeditions that collected study materials. SAK developed the scnDNA primers. DKC optimized DNA primers, and conducted the DNA sequencing. RCV, DKC, and SBJ completed the statistical analyses and prepared a final draft of the manuscript.

## Supplementary Material

Additional file 1 Non-degenerate and internal primer pairs (indicated with *) developed for population screening of *Riftia pachyptila*. GenBank accession numbers are indicated along with the PCR program (see text for details). A table of DNA primer pairs used in screening nuclear and mitochondrial genes in *R. pachyptila*. Click here for file

Additional file 2 **Allelic diversity in *Riftia pachyptila***. A table of allelic frequencies for nuclear and mitochondrial genes in *R. pachyptila*. DNA haplotypes for alleles are identified by their polymorphic sites. Click here for file
