# Supplementary material for: Genetic diversity and demographic instability in Riftia pachyptila tubeworms from eastern Pacific hydrothermal vents
Source: BMC Evol Biol. 2011 Apr 13;11:96. doi: 10.1186/1471-2148-11-96 (PMC3100261; doi:10.1186/1471-2148-11-96)
Supplement: Additional file 1 — Non-degenerate and internal primer pairs (indicated with *) developed for population screening of Riftia pachyptila. GenBank accession numbers are indicated along with the PCR program (see text for details). A table of DNA primer pairs used in screening nuclear and mitochondrial genes in R. pachyptila. [file 1471-2148-11-96-S1.DOC]

Additional file 1. Non-degenerate and internal primer pairs (indicated with *) developed for population screening of *Riftia pachyptila*. GenBank accession numbers are indicated along with the PCR program (see text for details).

| Primer | Primer sequence (3'–5' direction) | Acc. No. | PCR | Ref.^1^ |
| --- | --- | --- | --- | --- |
| Mitochondrial |  |  |  |  |
| Cytb_424F | GGWTAYGTWYTWCCWTGRGGWCARAT |  | *i* | 1 |
| Cytb_876R | GCRTAWGCRAAWARRAARTAYCAYTCWGG |  | *i* | 1 |
| ScnDNAs |  |  |  |  |
| Rpt46.1F | CCTCAGAAAGACGGGGTACACCTA | U68736 | *i* | 2 |
| Rpt46.1R | CGTATCCTACGAAGAATGCTGAGG | U68737 | *i* | 2 |
| Rpt84F | CAGCTCCGCATGGGTGGGCCGGTG |  | *i* | 2 |
| *Rpt84.1intF | GCTTACAATCACCGCTCCTT |  |  | 3 |
| Rpt84R | CGCCACAGACTACAGCAAGACGAA |  | *i* | 2 |
| *Rpt84.1intR | AGGGGACGACTAACGTGATG |  |  | 3 |
| Nuclear introns |  |  |  |  |
| ATPSαF | GAGCCMATGCAGACTGGTATTAAGGCYGT | AL023815 | *ii* | 4 |
| *ATPSαF | GAGCCCATGCAGACTGGTATTAAGGCCGT |  |  | 3 |
| ATPSαR | TTGAANCKCTTCTGGTTGATGATGGTGTC | AL023815 | *ii* | 4 |
| *ATPSαR | TTGAANCTCTTCTGGTTGATGATGGTGTC |  |  | 3 |

1. References: (1) Boore J, Brown W: Complete sequence of the mitochondrial DNA of the annelid worm *Lumbricus terrestris*. *Genetics* 1995, 141:305-319; (2) Steven A. Karl, GenBank acc. nos. U68732–U68750; (3) this study; (4) Jarman SN, Ward RD, Elliott NG: Oligonucleotide primers for PCR amplification of coelomate introns. *Mar Biotech* 2002, 4:347-355.
