# Supplementary material for: Genetic diversity and demographic instability in Riftia pachyptila tubeworms from eastern Pacific hydrothermal vents
Source: BMC Evol Biol. 2011 Apr 13;11:96. doi: 10.1186/1471-2148-11-96 (PMC3100261; doi:10.1186/1471-2148-11-96)
Supplement: Additional file 2 — Allelic diversity in Riftia pachyptila. A table of allelic frequencies for nuclear and mitochondrial genes in R. pachyptila. DNA haplotypes for alleles are identified by their polymorphic sites. [file 1471-2148-11-96-S2.DOCX]

Additional file 2: Allelic diversity in *Riftia pachyptila*.

|  | Localities | | | | | | | |
| --- | --- | --- | --- | --- | --- | --- | --- | --- |
| Locus (allele)^1^ | N27 | N21 | N9 | GAR | S7 | S11 | S17 | S32 |
| *Cytb n* | *33* | *44* | *10* | *17* | *17* | *18* | *14* | *9* |
| (1) GTCGAC | 1 | 0.886 | 0.900 | 0.176 | 0.882 | 0.944 | 1 | 1 |
| (2) A..... |  |  | 0.100 | 0.824 |  | 0.056 |  |  |
| (3) .C.... |  |  |  |  | 0.118 |  |  |  |
| (4) .....A |  | 0.068 |  |  |  |  |  |  |
| (5) ..T... |  | 0.023 |  |  |  |  |  |  |
| (6) ...T.. |  | 0.023 |  |  |  |  |  |  |
| *Rpt 46.1* 2*n* | *56* | *98* | *38* | *36* | *36* | *38* | *34* | *16* |
| (1) CTGAGG | 0.339 | 0.459 | 0.447 | 0.750 | 0.917 | 0.895 | 0.853 | 0.875 |
| (2) ..A... | 0.411 | 0.306 | 0.368 | 0.083 | 0.083 | 0.079 | 0.147 | 0.063 |
| (3) .....A | 0.232 | 0.204 | 0.108 | 0.200 |  |  |  |  |
| (4) .C.... |  |  |  |  |  |  |  | 0.063 |
| (5) ..A... |  | 0.031 |  |  |  |  |  |  |
| (6) T..... |  |  |  |  |  | 0.026 |  |  |
| (7) ....C. | 0.018 |  |  |  |  |  |  |  |
| *Rpt 84.1* 2*n* | *62* | *104* | *38* | *40* | *36* | *42* | *40* | *18* |
| (1) CGGGCCGGG | 0.371 | 0.442 | 0.684 | 0.750 | 0.694 | 0.929 | 0.875 | 0.944 |
| (2) .......A. | 0.468 | 0.385 | 0.211 |  | 0.083 |  |  |  |
| (3) ........A | 0.145 | 0.106 | 0.079 | 0.100 |  |  |  |  |
| (4) ...A..... |  |  |  |  | 0.222 | 0.071 | 0.125 | 0.056 |
| (5) .A....... |  | 0.019 | 0.026 |  |  |  |  |  |
| (6) G........ |  |  |  | 0.125 |  |  |  |  |
| (7) ......A.. |  |  |  | 0.050 |  |  |  |  |
| (8) .....T... |  | 0.038 |  |  |  |  |  |  |
| (9) ..T...... | 0.016 |  |  |  |  |  |  |  |
| (10) ....T.... |  | 0.010 |  |  |  |  |  |  |
| *ATPSα* 2*n* | *54* | *106* | *38* | *40* | *38* | *21* | *38* | *18* |
| (1) A | 0.407 | 0.755 | 0.921 | 0.700 | 1 | 1 | 1 | 1 |
| (2) G | 0.593 | 0.245 | 0.079 | 0.300 |  |  |  |  |

^1^ Allelic haplotypes identified by variable sites. Substitutions relative to 'leading' allele indicated in bold type.
